# Successful Pelvic Resection for Acetabular Hydatidosis

**DOI:** 10.1155/2017/9495783

**Published:** 2017-10-09

**Authors:** Canoville Daniel, Hannebicque Matthieu, Rochcongar Goulven, Michon Jocelyn, Dumaine Valérie, Hulet Christophe

**Affiliations:** ^1^CHU de Caen, 14000 Caen, France; ^2^Hôpital Cochin, APHP, 75014 Paris, France

## Abstract

**Background:**

Hydatidosis of the bone is a rare occurrence (0.9 to 2.5% of all localization of the disease). In those occurrences, the pelvic bone is the second most frequent localization. Curative treatment of pelvic bone hydatidosis is difficult and a consensus is yet to be found.

**Clinical Case:**

We report a case of hydatidosis of the ischium, extended to the homolateral hip. The patient was treated through hip resection using patient-specific cutting guides, followed by total hip reconstruction. Albendazole was administered to the patient for two months before the surgery and for three months following the surgery.

**Conclusions:**

In a young patient, hydatidosis of the pelvic bone can be treated with satisfying results through wide resection of the hip coupled with an antiparasitic treatment administered before and after the surgery. Prosthetic reconstruction, similar to what is done in cancer surgery, restores good functions.

## 1. Introduction

Echinococcosis is a parasitic disease caused by the larvae of* Echinococcus granulosus* [1]. Hydatidosis of the bone is very rare (0.9 to 2.5% of cases) [2, 3] and preferentially affects the spine and pelvic bones. Diagnosis is often delayed due to highly unspecific clinical signs and a very long clinical latency, which favours its local invasion. Curative treatment of the osseous form of the disease is not unequivocal [2, 4–10]. We report a case of hydatidosis of the ischium, spread to the homolateral hip joint. The patient was treated through resection of the left hemipelvis involving monobloc arthrectomy, followed by prosthetic reconstruction, coupled with treatment with Albendazole for two months before surgery and for three months after surgery. We then review the research literature, focusing on hydatid cystic lesions in the pelvis bones. Cases involving the spine, sacrum, and peripheral bones are not covered in this research.

## 2. Clinical Case

**Figure 1:**
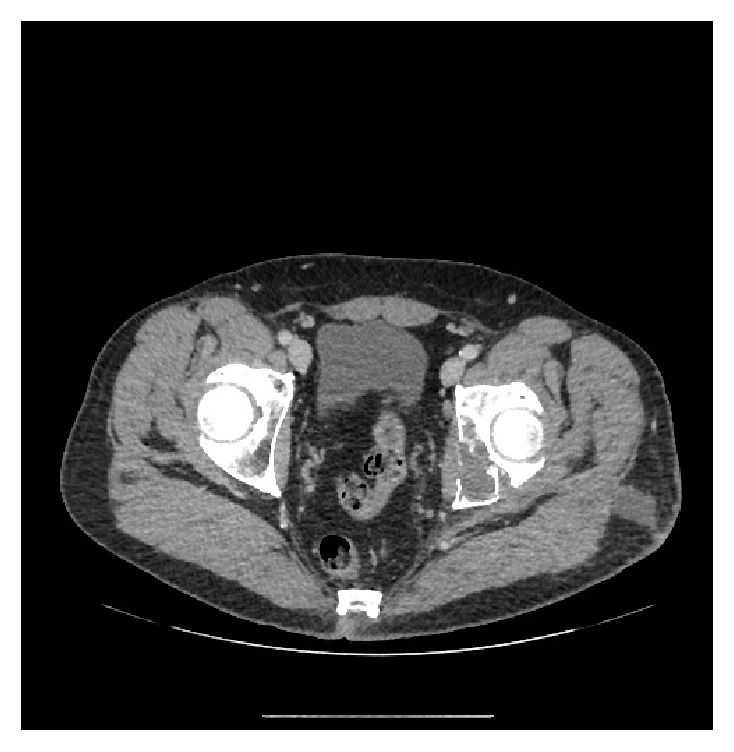
CT-scan: osteolytic lesion on the ischium.

**Figure 2:**
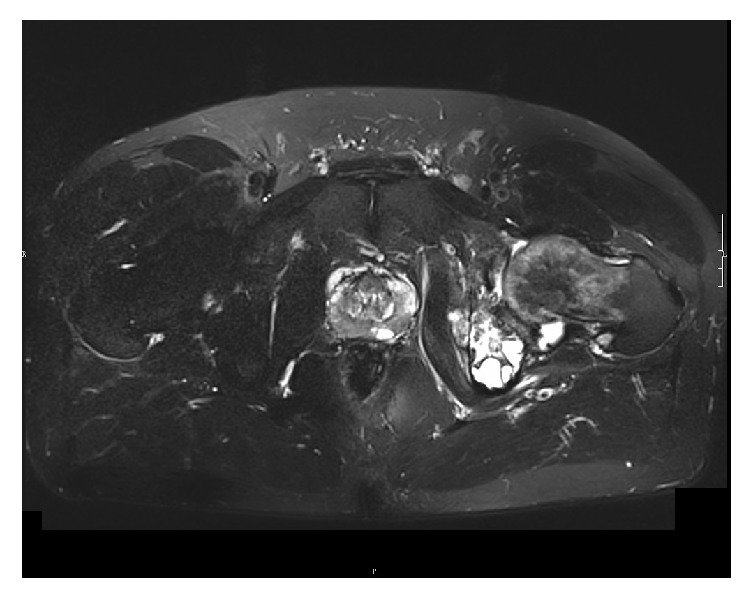
MRI: T2-weighted sequence.

The patient is a 55-year-old male who was first seen in May 2015 for inguinal pain which had progressively appeared in the previous 6 to 8 months. During clinical examination, the range of motion was preserved. The straight leg raise test was positive. No signs of biological or local inflammation were recorded. The complete blood count did not detect eosinophilia. Plain radiographs and CT-scan showed an osteolytic lesion on the ischiopubic branch invading the acetabulum (Figure 1). MRI confirmed the lesion and its extension to the left coxofemoral joint and to the obturator internus muscle (Figure 2), its heterogeneous nature, comprising both liquidous and tissular phases, and revealed a hypersignal inside the bone where the lesion laid. A thoracic-abdominal-pelvic CT-scan was carried out and found no other lesion.

A first CT-guided biopsy with fluid aspiration did not contribute to the diagnosis. A surgical biopsy was then carried out in June but also failed to contribute to the diagnosis, turning up amorphous material devoid of any cell. The biopsy was performed again in July, and the resulting anatomopathological analysis objectivised the hydatidosis on two fragments of necrotic tissue presenting hydatid fragments such as free scolex, hooks, and fragments of pellucid membrane.

We then started to administer Albendazole (800 mg per day in two doses) to the patient. This treatment was continued until surgery was carried out on the lesion. We then performed a resection of the left hemipelvis, with a monobloc arthrectomy performed because of the articular invasion. In order to facilitate the resection, we used patient-specific custom cutting guides that were made using fused CT-scan and MRI data (MOBELIFE®, Leuven, Belgium). The hip joint was then reconstructed with a total prosthesis combining an acetabular implant of the LUMIC (MUTARZ®) type and a cemented stem of the OCEANE (TORNIER®) type. The antiparasitic treatment was carried on for another three months.

**Figure 3:**
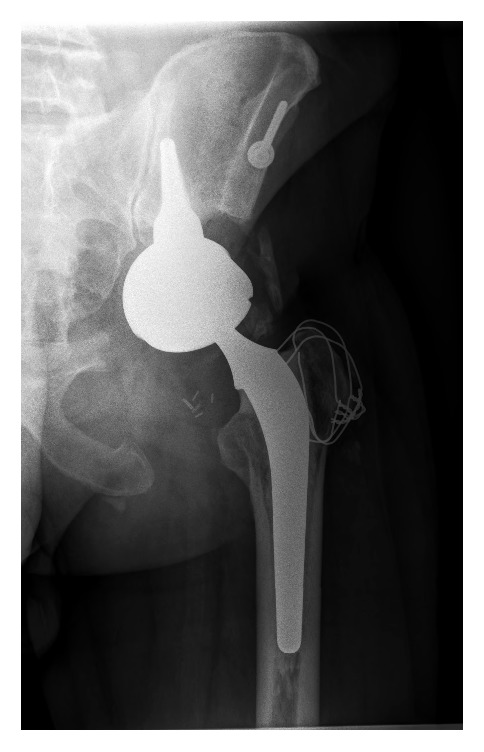
Radiographs: 12 months after surgery.

A year after surgery, our patient does not present signs of recurrence nor sepsis. He uses a walking aid outside his home (Harris Hip Score = 80.8 at 12 months). Control radiographs do not show any signs of unsealing on the stem and show good osseointegration of the acetabulum (Figure 3).

## 3. Discussion

We report the case of a 55-year-old male patient presenting hydatidosis of the left ischiopubic branch with a joint invasion. The disease, caused by* Echinococcus granulosus*, most frequently affects the liver (40%) and lungs (20%). Bone affection occurs in only 0.5% to 2% of cases, preferentially the spine (44%), the long bones (30%), the pelvis (16%), and then the flat bones. Bone contamination usually happens through haematogenous spread [2]. Simultaneous affection of the bones and viscera is a rare occurrence [2, 11, 12]. It was not present in our patient at the time of assessing CT-scan. Primitive localization on the pelvis is usually at the ileum.

The disease progresses step by step inside the bone and spreads regionally either via the joint connections to the sacrum or femur or due to a hydatid ossifluent abscess that develops from a pathologic fracture or a cortical effraction [2]. We can assume that our patient started to experience pain when the disease progressed to the coxofemoral joint, from an initial lesion in the ischium that had been developing from an earlier time.

We had an 8 to 10 months' diagnosis latency on this case, starting from the time when the pain first appeared. Diagnosis latency is usually long for the pelvic forms of the disease, Markakis et al. [13] having recorded 28 months of latency. In most cases, osseous hydatidosis clinical signs are pain or a cold abscess when neighbouring soft tissues are affected. A history of infection contact or a history of visceral echinococcosis can guide the diagnosis. In our case, the patient had travelled to Dubai once a year since 2009 but not to a risk zone. Anamnesis did not either bring up any stay in the Mediterranean basin, which is an endemic zone of echinococcosis. Biological testing of our patient did not turn up eosinophilia, which only appears in 25% of cases [14]. Serological tests, which were not carried out in this case, usually contribute to the diagnosis to a lesser degree in the case of bone affection than in the case of visceral affection [2, 15].

Images obtained through plain radiography were lacunar and aspecific. The classic “honeycomb” pattern was not as obvious here as in other cases where local extension is more significant [6, 16]. However, the lack of peripheral osseous sclerosis and the lack of periosteal reaction both support the diagnosis [2, 17]. The CT-scan allows for a finer analysis of the osteolysis, which is diffuse and not clearly delimited. MRI scanning remains the method of choice to evaluate the local and regional extension [2, 18]. The vesicles appear to be liquidous, showing as a hypersignal on the T2-weighted sequences and a hyposignal on the T1-weighted sequences, not gadolinium enhancing. Lesions can take a pseudotumoral appearance, especially when the septa are hypointense on T1 and hyperintense on T2.

The surgical treatment of lesions is not unequivocal, as shown by the diversity of the modalities of treatment that have been experimented and reported in the literature. There is a high rate of recurrence after partial resection [2, 19, 20]. However, Martínez et al. report several cases where limited resection surgery was used with satisfying results to treat purely osseous affections [10]. In this series, when the joint was involved, treating with only a curettage operation yielded 100% recurrence. The approach to curative treatment of the lesions usually accepted nowadays combines a wide resection with an antiparasitic treatment before and after surgery [2, 5–7, 16, 21]. This pushed us to carry out a monobloc resection and arthrectomy followed by a reconstruction consisting in total hip replacement. We chose to use patient-specific, single-use cutting guides, which have already been proven to be useful for oncologic surgery [22]. We favoured this solution for our young, active patient over arthrodesis or Girdle arthroplasty, which induce very significant function restriction, and over other treatments which are still in an experimental stage [5, 6, 16]. Moreover, this procedure is also carried out in reconstructions following the resection of malignant lesions and is therefore well understood.

Medicinal antiparasitic treatment is recommended before and after surgery according to WHO [2, 23–25]. We then treated our patient accordingly. Such a treatment helps to sterilise the cysts before surgery and to reduce their size. Albendazole is recommended at a dose of 10 to 15 mg/kg/day. This is meant to reduce the risk of recurrence. However, the follow-up period is short in our case and recurrence might still occur, thus requiring frequent follow-up testing and radiographs.

## 4. Conclusions

In a young patient, hydatidosis of the pelvis and hip can be treated with satisfying results through wide resection of the hip bone coupled with an antiparasitic treatment administered before and after surgery. Prosthetic reconstruction, similar to what is done in oncologic surgery, restores good functions.
