# Corrigendum to “Successful Pelvic Resection for Acetabular Hydatidosis”

**DOI:** 10.1155/2019/2942858

**Published:** 2019-09-04

**Authors:** Daniel Canoville, Matthieu Hannebicque, Goulven Rochcongar, Jocelyn Michon, Valérie Dumaine, Christophe Hulet

**Affiliations:** ^1^CHU de Caen, 14000 Caen, France; ^2^Hôpital Cochin, APHP, 75014 Paris, France

In the article titled “Successful Pelvic Resection for Acetabular Hydatidosis” [1], there was an error in the authors' name format as they were reversed. The correct author list and affiliations are shown above.
